# Transcriptomic characterization of two major *Fusarium* resistance quantitative trait loci (QTLs), *Fhb1* and *Qfhs.ifa‐5A*, identifies novel candidate genes

**DOI:** 10.1111/mpp.12048

**Published:** 2013-06-06

**Authors:** Wolfgang Schweiger, Barbara Steiner, Christian Ametz, Gerald Siegwart, Gerlinde Wiesenberger, Franz Berthiller, Marc Lemmens, Haiyan Jia, Gerhard Adam, Gary J. Muehlbauer, David P. Kreil, Hermann Buerstmayr

**Affiliations:** ^1^ Institute for Biotechnology in Plant Production University of Natural Resources and Life Sciences Konrad Lorenz Straße 20 3430 Tulln Austria; ^2^ Department of Applied Genetics and Cell Biology University of Natural Resources and Life Sciences Konrad Lorenz Straße 24 3430 Tulln Austria; ^3^ Christian Doppler Laboratory for Mycotoxin Metabolism IFA‐Tulln University of Natural Resources and Life Sciences Konrad Lorenz Straße 20 3430 Tulln Austria; ^4^ Department of Agronomy and Plant Genetics Department of Plant Biology University of Minnesota St. Paul MN 55108 USA; ^5^ Department of Biotechnology University of Natural Resources and Life Sciences Muthgasse 18 1190 Vienna Austria; ^6^ School of Life Sciences University of Warwick Coventry CV4 7AL UK

## Abstract

Fusarium head blight, caused by *Fusarium graminearum*, is a devastating disease of wheat. We developed near‐isogenic lines (NILs) differing in the two strongest known *F. graminearum* resistance quantitative trait loci (QTLs), *Qfhs.ndsu‐3BS* (also known as resistance gene *Fhb1*) and *Qfhs.ifa‐5A*, which are located on the short arm of chromosome 3B and on chromosome 5A, respectively. These NILs showing different levels of resistance were used to identify transcripts that are changed significantly in a QTL‐specific manner in response to the pathogen and between mock‐inoculated samples. After inoculation with *F. graminearum* spores, 16 transcripts showed a significantly different response for *Fhb1* and 352 for *Qfhs.ifa‐5A*. Notably, we identified a lipid transfer protein which is constitutively at least 50‐fold more abundant in plants carrying the resistant allele of *Qfhs.ifa‐5A*. In addition to this candidate gene associated with *Qfhs.ifa‐5A*, we identified a uridine diphosphate (UDP)‐glycosyltransferase gene, designated *TaUGT12887*, exhibiting a positive difference in response to the pathogen in lines harbouring both QTLs relative to lines carrying only the *Qfhs.ifa‐5A* resistance allele, suggesting *Fhb1* dependence of this transcript. Yet, this dependence was observed only in the NIL with already higher basal resistance. The complete cDNA of *TaUGT12887* was reconstituted from available wheat genomic sequences, and a synthetic recoded gene was expressed in a toxin‐sensitive strain of *Saccharomyces cerevisiae*. This gene conferred deoxynivalenol resistance, albeit much weaker than that observed with the previously characterized barley HvUGT13248.

## Introduction

The plant‐pathogenic fungus *Fusarium graminearum* frequently infects small grain cereals, particularly wheat (Leonard and Bushnell, 2003). Fusarium head blight (FHB) is one of the most important field crop diseases in temperate regions, resulting in severe yield losses and reduced grain quality by contamination with the trichothecene deoxynivalenol (DON; Desjardins, 2006). This mycotoxin is a serious threat to human and animal health (Pestka, 2010). Maximum tolerated levels in food have been enacted in the European Union (The European Commission, 2006), and advisory levels have been issued by the Food and Drug Administration in the USA. As the control of FHB by crop rotation, tillage regimes or the use of fungicides is only partially successful, an understanding of the genetic control of FHB resistance and the deployment of resistance genes in wheat varieties are likely to be the most promising strategies.

Genetic mapping of FHB resistance in wheat has led to the identification of several quantitative trait loci (QTLs) that confer partial resistance (Buerstmayr *et al*., 2009). The two most effective QTLs exhibiting FHB resistance were identified in the Chinese Spring wheat cultivar Sumai3 and derivatives, and are positioned on the short arm of chromosome 3B (*Fhb1*) and on chromosome 5A (*Qfhs.ifa‐5A*) (Anderson *et al*., 2001; Buerstmayr *et al*., 2002, 2003). Molecular markers have been developed for the accurate screening of *Fhb1* (Liu *et al*., 2008) and *Qfhs.ifa‐5A* (Buerstmayr *et al*., 2002) resistance alleles. *Fhb1* and *Qfhs.ifa‐5A* employ different mechanisms. *Fhb1* confers so‐called type II resistance by slowing down or inhibiting the spread of the pathogen from the initial infection site, whereas *Qfhs.ifa‐5A* contributes to type I resistance by lowering the rate of initial infection and also confers type II resistance – albeit to a lesser extent.

The genetic determinants underlying *Fhb1* and *Qfhs.ifa‐5A* are still unknown. Despite significant efforts, the *Fhb1* gene has not yet been isolated (Liu *et al*., 2006, 2008). In addition, the release of parts of the wheat chromosome 3BS sequence of the FHB‐susceptible cultivar Chinese Spring, which should cover the *Fhb1* locus, has not led to new insights into the genetic determinants of *Fhb1* (Choulet *et al*., 2010).

Previous work has indicated that *Fhb1* plays a role in resistance against DON, a major virulence factor for *F. graminearum*. Lemmens *et al*. (2005) have shown that lines carrying the *Fhb1* resistant allele exhibit increased ability to conjugate DON into DON‐3‐glucoside (D3G). A highly DON‐inducible barley uridine diphosphate‐glycosyltransferase (UGT), capable of inactivating DON by transforming it to D3G, has been identified (Gardiner *et al*., 2010; Schweiger *et al*., 2010), and transgenic Arabidopsis carrying the barley UGT exhibits high DON resistance (Shin *et al*., 2012).

In contrast, a recent report has suggested that high levels of phenylpropanoids observed in *F. graminearum*‐inoculated spikelets are responsible for the resistance conferred by *Fhb1*, which may be related to the also observed higher abundance of the biotic stress hormone jasmonic acid (JA; Gunnaiah *et al*., 2012). In addition, Xiao *et al*. (2013) have identified JA signalling, but not biosynthesis, as specific for *Fhb1*‐related resistance. Yet, this is difficult to reconcile with the results of studies using pure toxin (Horevaj *et al*., 2012; Lemmens *et al*., 2005). Possibly, in heavily inoculated spikelets, resistance is quickly overcome before the induction and function of UGT. Thus, induced UGT activity may be required for the inactivation of DON diffusion ahead of hyphae into the surrounding tissue. Alternatively, Zhuang *et al*. (2013) have associated the downregulation of a pectin methyl esterase inhibitor gene in strains lacking *Fhb1* with the QTL. Overexpression in durum wheat of such a gene from *Actinidia chinensis* led to a reduction in FHB symptoms (Volpi *et al*., 2011). In addition, pathogenesis‐related proteins and an ABC transporter have been found to be more abundant for the QTL (Gunnaiah *et al*., 2012), and an Rpg1 kinase homologue was mapped to the locus (Shen *et al*., 2006).

This report investigates transcriptional differences of near‐isogenic lines (NILs) segregating for *Fhb1*, and is the first to investigate *Qfhs.ifa‐5A*. We report a candidate gene – a lipid transfer protein (LTP) constitutively more abundant for the resistant *Qfhs.ifa‐5A* allele. A UGT gene more responsive in the *Fhb1* background – if *Qfhs.ifa‐5A* is also present – was identified and functionally tested by heterologous expression in yeast.

## Results

### Resistance QTLs in a near‐isogenic background confer different resistance levels

**Figure 1:**
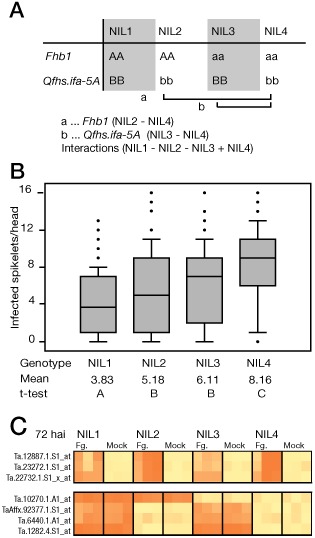
Characterization of near‐isogenic lines (NILs) generated in this study. (A) Each NIL harbours a different composition of resistant (AA, BB) or susceptible (aa, bb) alleles of *Fhb1* and *Qfhs.ifa‐5A*. The *Fusarium graminearum*‐specific responses for *Fhb1* and *Qfhs.ifa‐5A* were calculated by comparing the *F. graminearum*/mock contrasts of the indicated lines. Differences in constitutive gene expression were calculated by comparing mock‐inoculated samples of the same lines. The ‘interaction’ contrast indicates nonadditive effects between *Fhb1* and *Qfhs.ifa‐5A* in NIL1 harbouring both resistance alleles. (B) Comparison of disease progression observed 18 days after inoculation in each of the NILs. (C) Heat map showing differential accumulation of exemplary transcripts between NILs at 72 h after inoculation (hai). The top panel represents *Fusarium*‐responsive transcripts: Ta.12887.1.S1_at, Ta.23272.1.S1_at and Ta.22732.1.S1_at (all significantly changed for *Qfhs.ifa‐5A*). The bottom panel represents transcripts with constitutive differences in abundance: TaAffx.92377.1.S1_at, Ta.6440.1.A1_at, Ta.1282.4.S1_at (all *Qfhs.ifa‐5A*) and Ta.10270.1.A1_at (*Fhb1*).

In an effort to identify genetic determinants underlying two validated FHB resistance QTLs in wheat, *Fhb1* and *Qfhs.ifa‐5A*, we generated NILs from a cross of the susceptible spring wheat cv. Remus and the FHB resistant line CM‐82036, carrying the *Fhb1* and *Qfhs.ifa‐5A* QTLs. Four NILs from a BC_5_F_2_ population were selected for the presence of the resistant alleles at both QTLs (NIL1), harbouring only the *Fhb1* (NIL2) or only the *Qfhs.ifa.5A* (NIL3) resistance allele, or carrying the susceptible alleles at both QTLs (NIL4) (Fig. 1A). Diversity Arrays Technology (DArT) marker analysis showed that the percentage of Remus alleles varied between 96.3% (NIL1) and 99.8% (NIL4).

To phenotypically assess the resistance levels, 150 heads per genotype were used to estimate disease progression, 12, 18 and 24 days after point inoculation of single spikelets with *F. graminearum* spores. NIL4 developed a similar phenotype to the susceptible cv. Remus, and NIL1 exhibited the highest level of resistance among NILs, but did not perform as well as the parent CM‐82036, which developed, on average, only one diseased spikelet (not shown). Intermediate resistance levels were observed in NIL2 (*Fhb1*) and NIL3 (*Qfhs.ifa‐5A*) (*t*‐test, *P* ≤ 0.05; Fig. 1B). At maturity, kernels of whole wheat ears were investigated for DON and D3G content, which confirmed the phenotyping results. The more resistant line NIL1 exhibited the lowest DON content (on average 9.45 mg/kg) and the highest D3G/DON ratio (17%); NIL2 and NIL3 contained, on average, 20.25 mg/kg and 18.52 mg/kg DON, respectively, and ratios of 12% and 14% D3G/DON. The susceptible NIL4 showed the highest DON content (41.35 mg/kg) and the lowest D3G/DON ratio (8%).

However, inoculated samples from the time points used for the transcriptome analysis [72 h after inoculation (hai) and earlier] contained only trace amounts of DON.

### Transcriptional characterization of the wheat response to *F. graminearum*

We assessed QTL‐specific differential transcript abundance between the four NILs related to treatment (*F. graminearum* vs. mock) and time point (8, 24 and 72 hai) in three individual replicates using the Affymetrix 61 K wheat GeneChip. Several examples are shown in Fig. 1C. In total, we identified 806 transcripts responding to *F. graminearum* infection in at least one time point when calculating the average (i.e. genotype‐independent) differences in transcript abundance between *F. graminearum*‐ and mock‐inoculated samples (adjusted *P* ≤ 0.05). The vast majority of these transcripts (792) were detected at 72 hai, less at 24 hai (62) and a few at 8 hai (29). The *Fhb1*‐ and *Qfhs.ifa‐5A*‐specific responses were generated by subtracting the *F. graminearum*/mock contrast of NIL4 (susceptible QTL alleles) from either NIL2 (resistant *Fhb1* allele) or NIL3 (resistant *Qfhs.ifa‐5A* allele) (Fig. 1A). The majority of transcripts identified with these contrasts showed a negative difference in response to *F. graminearum*, indicating a higher transcript accumulation in the presence of the susceptible QTL alleles (Table S1, see Supporting Information). We confirmed the observed differences by reverse transcription‐quantitative polymerase chain reaction (RT‐qPCR) for a representative group of probe sets showing the highest relative changes. The investigated transcripts comprise *F. graminearum*‐induced and QTL‐specific genes (Fig. S1A, see Supporting Information), and genes which are constitutively changed between mock‐treated lines for one of the QTLs (Fig. S1B). Potentially, observed transcript levels can be confounded by single‐feature polymorphisms (SFPs) that lead to different binding affinities to the probe sets. However, such SFPs were detected for only a few transcripts. Transcripts were functionally annotated and compared against available databases, and with annotations reported previously by Jia *et al*. (2009). For ease of comparison with Jia *et al*. (2009), we classified the annotated genes by their putative roles as related to defence. In total, we identified 207 genes with a proposed role in defence and 264 involved in other categories (Table S2, see Supporting Information).

### Broad and late defence mechanisms are mounted in the absence of the *Qfhs.ifa‐5A* QTL

The contrast to detect *Qfhs.ifa‐5A* gene‐specific expression was calculated by subtracting the *F. graminearum*/mock contrast of NIL4 from the *F. graminearum*/mock contrast of NIL3 with the resistant *Qfhs.ifa‐5A* allele, and detected 352 transcripts as differently regulated. Of these, 339 were detected at 72 hai and, with one exception (Ta.9975.2.S1_at, unknown), all showed a negative difference in response, indicating a higher induction or more sustained expression in the more susceptible genotype lacking *Qfhs‐ifa.5A* resistance. These 338 genes (Table S3, see Supporting Information) encode a variety of defence‐related proteins.

Twenty‐one probe sets correspond to genes involved in the phenylpropanoid pathway, including multiple transcripts encoding phenylalanine ammonia lyases (PALs), the first dedicated step in the pathway. Only two more genes in this pathway that were not *Qfhs.ifa‐5A* specific were identified when calculating the average differences in transcript abundance between *F. graminearum*‐ and mock‐inoculated samples across all lines. Most of the identified transcripts involved in the production of ethylene (ET) and JA showed higher accumulation for the susceptible *Qfhs.ifa‐5A* allele (one and nine transcripts, respectively). Similarly, *F. graminearum*‐responsive transcripts potentially involved in DON detoxification were found to be predominantly more abundant in lines harbouring the susceptible *Qfhs.ifa‐5A* allele. These transcripts correspond to 12 UGTs (from a total of 14 UGTs detected), three MATE‐type transporter genes (three) and nine pleiotropic drug resistance transporter genes (18).

In contrast, transcripts corresponding to pathogenesis‐related proteins were, in most cases, detected as a general response to the pathogen and not by the *Qfhs.ifa‐5A*‐specific contrast. These included the subclasses of chitinases, glucanases, thaumatin and thaumatin‐like proteins, as well as LTPs, which are involved in the early interaction with the pathogen (Walter *et al*., 2010). However, the expression of germin‐like proteins and protease inhibitors was almost exclusively detected in the more vulnerable line carrying the susceptible *Qfhs.ifa‐5A* allele (see also Table S2).

At time points other than 72 hai, five probe sets with a positive difference in response to *F. graminearum* were identified for *Qfhs.ifa‐5A* (8 hai), indicating higher accumulation for the resistant allele. These encoded a stress‐responsive A/B barrel domain‐containing protein, a calmodulin‐related calcium sensor protein, a glutathione‐*S*‐transferase (GST), a nonsymbiotic haemoglobin and one unknown protein. Notably, two of these genes also exhibited a negative difference at 72 hai, with higher accumulation in the genotype with the susceptible *Qfhs.ifa‐5A* allele (Ta.5610.1.S1_at, nonsymbiotic haemoglobin 2 and TaAffx.112045.1.S1_x_at, GST). *Fusarium graminearum*‐responsive transcripts exhibiting a significant difference in response for *Qfhs.ifa‐5A* are summarized in Table 1.

**Table 1:** Transcripts exhibiting a differential response for *Qfhs.ifa‐5A* a (adjusted *P* ≤ 0.05, ≥2 fold change, FC) after inoculation with *Fusarium graminearum* spores

Affymetrix ID	Annotation^b^	Classification	FC, 8 hai^c^	FC, 24 hai	FC, 72 hai	Significant in other contrasts^d^	Mapped to^e^
Ta.9975.2.S1_at	Unknown	Unknown	1.51	0.95	**6.28**		
Ta.14573.1.S1_at	Hsp20/α‐crystallin family protein (*Oryza sativa*)	Defence	**0.23**	0.80	1.68		
Ta.13859.1.A1_at	OsCML16; calmodulin‐related calcium sensor protein (*Oryza sativa*)	Regulatory	**2.94**	0.64	1.30	−*Fhb1* (24 hai)	
Ta.16827.1.S1_s_at	Unknown	Unknown	0.75	**0.35**	1.25		
Ta.10311.1.S1_at	Stress‐responsive A/B barrel domain‐containing protein (*Oryza sativa*)	Unknown	**2.65**	1.01	1.16		
Ta.13426.1.S1_s_at	Os04g0119500 protein (*Oryza sativa*) (UniRef90_Q7XTJ2)	Unknown	**3.14**	0.89	1.09		
TaAffx.64985.1.S1_at	Putative uncharacterized protein (*Zea mays*) (UniRef90_B4FQP2)	Regulatory	**0.17**	2.02	1.00		
Ta.9389.1.S1_at	Seed maturation protein PM41 (*Oryza sativa*)	Development	2.20	**0.31**	0.98		
Ta.20205.1.A1_s_at	VER2 (*Triticum aestivum*) (UniRef90_O80370)	Development	1.14	**0.48**	0.95		
Ta.24801.1.S1_at	Hsp20/α‐crystallin family protein (*Oryza sativa*)	Defence	**0.25**	0.95	0.90		
Ta.5004.1.S1_at	Thiol protease SEN102 precursor (*Oryza sativa*)	Metabolism	**0.26**	1.70	0.89	−*Fhb1* (8 hai)	
Ta.5004.3.S1_a_at	Thiol protease SEN102 precursor (*Oryza sativa*)	Metabolism	**0.47**	1.19	0.88	−*Fhb1* (8 hai)	
TaAffx.112045.1.S1_x_at	Glutathione‐*S*‐transferase (*Oryza sativa*)	Defence	**3.20**	1.23	**0.03**	+*Fhb1* (8 hai)	
Ta.5610.1.S1_at	Nonsymbiotic haemoglobin 2 (*Oryza sativa*)	Miscellaneous	**7.01**	0.40	**0.02**		4BL, 4DL

a The expression ratio was calculated from the *F. graminearum*/mock response observed in NIL3 (resistant *Qfhs.ifa‐5A* allele) and the response provided by NIL4 (susceptible allele). Genes more abundant in the absence of *Qfhs.ifa‐5A* at 72 hai are excluded here and provided in Table S3 (see Supporting Information).

b Annotations were assigned if the *E* value from the BlastX results was ≤10^−10^; otherwise the transcripts were denominated as ‘unknown’.

c Differences in response are the means of three biological replicates. Statistically significant values are given in bold type.

d −/+ indicates significant positive/negative differences in response for the indicated contrast.

e Map locations were identified by searching the mapped expressed sequence tag (EST) database of Graingenes for the probe set sequences using BlastN and an *E* cut‐off of ≤10^−10^.

### An LTP is constitutively more abundant in the presence of *Qfhs.ifa‐5A*

Twenty‐eight genes were constitutively more and 63 less abundant in the presence of the resistant allele when comparing mock‐inoculated samples of NIL3 and NIL4 differing in *Qfhs.ifa‐5A* (Table S4, see Supporting Information, and Table 2, which summarizes the group of more abundant transcripts). Of these, 16 probe sets corresponded to defence‐related genes, including three heat shock proteins more abundant only at 8 hai (two isogenes) or 72 hai (one isogene). The largest difference in transcript abundance was observed for Ta.1282.4.S1_at, which corresponds to an LTP. The probe set derives from an expressed sequence tag (EST) (GenBank accession CA635994), which is identical to the gene model of TaLTP9.4a (GenBank accession AJ852541, cv. Apache; Boutrot *et al*., 2007). We used specific primers for the 5′ untranslated region (UTR) published for TaLTP9.4a to map Ta.1282.4.S1_at to the long arm of chromosome 5A using a collection of Chinese Spring nulli‐tetra lines. Deletion bin mapping of this gene using four Chinese Spring deletion lines for 5AS and five deletion lines for 5AL produced the specific PCR fragment for all lines investigated, suggesting that this gene is located on the pericentromeric region of 5AL. The gene model of this LTP contains one 440‐bp intron, which separates the 369‐bp coding sequence (CDS) into two exons of 360 and 9 bp. Resequencing of cloned PCR products from both the resistant CM‐82036 and the susceptible Remus identified two highly similar LTP genes, which exist in both genotypes. These genes, potentially paralogues positioned on the same locus, differ in two and six single‐nucleotide polymorphisms (SNPs), respectively, from the EST template (Fig. S2, see Supporting Information).

**Table 2:** Transcripts constitutively more abundant (adjusted *P* ≤ 0.05, ≥2 fold change, FC) in the presence of the resistance alleles of either *Fhb1* or *Qfhs.ifa‐5A* a

Affymetrix ID	Annotation^b^	Classification	FC, 8 hai^c^	FC, 24 hai	FC, 72 hai	SFP^d^	Mapped to^e^
Constitutively changed for *Fhb1*							
Ta.28411.1.A1_at	OsCML16; calmodulin‐related calcium sensor protein (*Oryza sativa*)	Regulatory	**33.53**	**95.9**	**53.74**	11	
Ta.22694.1.A1_at	Unknown	Unknown	**30.91**	**21.96**	**36.91**	5	
Ta.13161.1.S1_at	Putative extensin‐like cell wall protein (Jia *et al*., 2009)	Cell wall	**21.24**	**23.13**	**36.24**	8, 9	
Ta.10270.2.A1_a_at	Wax synthase (*Oryza sativa*)	Metabolism	**5.62**	**6.42**	**6.1**		
Ta.10270.1.A1_at	Putative uncharacterized protein Sb02g001860 (*Sorghum bicolor*) (UniRef90_C5X8N1)	Unknown	**5.15**	**5.18**	**4.94**		
TaAffx.654.1.S1_at	Unknown	Unknown	**2.89**	**2.65**	**2.99**		5DL
TaAffx.12498.2.S1_at	Unknown	Unknown	**2.54**	**2.41**	**2.84**		
Ta.5633.1.S1_a_at	Unknown	Unknown	1.02	**3.76**	2.7		
Ta.25791.1.A1_at	Unknown	Unknown	**3.15**	**4.83**	**2.55**	10	
TaAffx.130179.1.A1_at	Putative uncharacterized protein (*Oryza sativa* Indica Group) (UniRef90_A2YVE1)	Unknown	1.86	**2.29**	**2.55**		
TaAffx.86734.1.S1_at	Unknown	Unknown	**2.14**	**2.11**	**2.06**		
TaAffx.12498.1.S1_s_at	Unknown	Unknown	**2.13**	1.99	1.89		
Ta.13263.2.A1_a_at	Unknown	Unknown	1.39	**2.25**	1.39		
Ta.9389.1.S1_x_at	Seed maturation protein PM41 (*Oryza sativa*)	Development	**0.33**	**2.67**	1.2		
TaAffx.616.2.S1_s_at	Hypothetical protein (Jia *et al*., 2009)	Unknown	0.58	**2.83**	1.12		
Ta.28659.3.S1_x_at	Subtilisin–chymotrypsin inhibitor 2 (Jia *et al*., 2009)	Defence	**0.44**	**3.54**	1.05		
Ta.9389.1.S1_at	Seed maturation protein PM41 (*Oryza sativa*)	Development	**0.3**	**2.44**	1.04		
Constitutively changed for *Qfhs.ifa‐5A*							
Ta.1282.4.S1_at	Nonspecific lipid transfer protein (Triticeae) (UniRef90_Q42848)	Defence	**51.75**	**70.04**	**65.85**	4, 6	
Ta.6440.1.A1_at	Os09g0436400 protein (*Oryza sativa* Japonica Group) (UniRef90_Q0J1J3)	Unknown	**34.52**	**29.3**	**41.21**	8	
Ta.27806.1.A1_at	Unknown	Unknown	**5**	**13**	**21**		
TaAffx.68700.1.S1_at	Expressed protein (*Oryza sativa*)	Unknown	**9.92**	**19.24**	**17.4**		
TaAffx.36760.1.S1_at	RCN1 Centroradialis‐like1 homologous to TFL1 gene; contains Pfam profile PF01161: phosphatidylethanolamine‐binding (*Oryza sativa*)	Regulatory	3.2	**6.31**	**7.26**		2AL, 2DL, 4AL
Ta.11043.1.A1_at	Unknown	Unknown	**5.28**	**6.67**	**5.8**		
TaAffx.70660.1.S1_at	Hsp20/α‐crystallin family protein (*Oryza sativa*)	Defence	1.5	1.03	**4.77**		
TaAffx.92377.1.S1_at	Predicted protein (*Populus trichocarpa*) (UniRef90_B9GJ95)	Regulatory	**3.74**	**4.23**	**4.41**		
TaAffx.9181.1.S1_at	Unknown	Unknown	**3.61**	**3.13**	**4.19**	7	
Ta.5776.1.S1_at	Unknown	Unknown	0.96	1.03	**3.81**		
Ta.25955.1.A1_at	Unknown	Unknown	**2.7**	1.93	**2.82**		
Ta.23102.1.S1_at	HEAT repeat family protein (*Oryza sativa*)	Miscellaneous	**5.4**	2.37	2.64		2DS, 5AS, 5BS, 5DS
TaAffx.23477.1.S1_at	Unknown	Unknown	**2.53**	**2.62**	**2.53**		
Ta.6352.3.A1_a_at	XH domain‐containing protein (*Oryza sativa*)	Regulatory	**2.42**	**2.7**	**2.51**		
TaAffx.86453.1.S1_s_at	Unknown	Unknown	1.92	**2.89**	2.24		
Ta.5597.1.S1_at	Auxin‐repressed protein (*Oryza sativa*)	Unknown	1.62	1.72	**2.15**	7	4DL, 6BS
Ta.12038.1.A1_at	MDR‐like ABC transporter (*Oryza sativa*)	Putative trichothecene detoxification and transport	1.86	**2.13**	**2.06**		
Ta.5281.1.S1_at	Hydrolase, α/β fold family domain‐containing protein (*Oryza sativa*)	Metabolism	**3.42**	3.91	1.85		
Ta.4381.2.S1_a_at	Unknown	Unknown	**2.5**	**2.13**	1.74		7AS
Ta.29578.2.S1_at	Nonsymbiotic haemoglobin 2 (*Oryza sativa*)	Miscellaneous	0.84	**2.06**	1.7		
Ta.867.1.A1_at	Nucleotide pyrophosphatase/phosphodiesterase (Andropogoneae) (UniRef90_B6U857)	Metabolism	**2.39**	**2.41**	1.68		
Ta.13156.1.S1_at	Unknown	Unknown	**2.13**	1.96	1.64		
Ta.8274.1.A1_at	Unknown	Unknown	**2.37**	**2.26**	1.56		
Ta.540.1.S1_at	Unknown	Unknown	**2.02**	1.34	1.53		1AL, 3AS
Ta.19896.1.A1_at	Os09g0425200 protein (*Oryza sativa*) (UniRef90_Q69PC6)	Unknown	**2.24**	1.48	1.36		
Ta.24801.1.S1_at	Hsp20/α‐crystallin family protein (*Oryza sativa*)	Defence	**4.55**	1.03	1.36		
Ta.11111.1.A1_at	RCN4 Centroradialis‐like1 homologous to TFL1 gene (*Oryza sativa*)	Development	**2.92**	1.81	1		
Ta.14573.1.S1_at	Hsp20/α‐crystallin family protein (*Oryza sativa*)	Defence	**4.77**	1.29	0.86		

a The expression ratios were calculated by comparing mock‐treated samples of NIL2 (resistant *Fhb1* allele) or NIL3 (resistant *Qfhs.ifa‐5A* allele) with NIL4 (susceptible alleles).

b Annotations were assigned if *E* from the BlastX results was ≤10^−10^; otherwise, the transcripts were denominated as ‘unknown’.

c Differences in transcript abundance are the means of three biological replicates. Statistically significant values are given in bold type.

d Numbers in the single‐feature polymorphism (SFP) column indicate for which probes SFPs were detected.

e Map locations were identified by searching the mapped expressed sequence tag (EST) database of Graingenes for the probe set sequences using BlastN and a *E* ≤ 10^−10^.

### *Fusarium graminearum*‐responsive *Fhb1*‐specific transcripts

The *F. graminearum*‐specific and *Fhb1*‐dependent response was generated by subtracting the *F. graminearum/*mock contrast of NIL4 (both susceptible QTL alleles) from the *F. graminearum/*mock contrast derived from NIL2 (resistant *Fhb1* allele). We identified 16 transcripts exhibiting a differential response for *Fhb1* (Table 3). At 8 hai, only one transcript exhibited a positive difference in response (TaAffx.112045.1.S1_x_at; GST), and two probe sets had a negative difference in response (Ta.5004.1.S1_at; Ta.5004.1.S1_x_at; thiol protease precursors). With one exception (Ta.14071.1.S1_x_at; integral membrane protein), all of the transcripts identified at 72 hai (12) and 24 hai (two) showed a negative difference in response, indicating less accumulation in the line carrying the resistant *Fhb1* allele compared with the line carrying the susceptible allele.

**Table 3:** Transcripts exhibiting a differential response for *Fhb1* a (adjusted *P* ≤ 0.05, ≥2 fold change, FC) after inoculation with *Fusarium graminearum* spores

Affymetrix ID	Annotation^b^	Classification	FC, 8 hai^c^	FC, 24 hai	FC, 72 hai	Significant in other contrasts^d^	Mapped to^e^
Ta.14071.1.S1_x_at	Integral membrane protein DUF6‐containing protein, expressed (*Oryza sativa*)	Metabolism	1.37	1.24	**3.17**		
Ta.13859.1.A1_at	OsCML16; calmodulin‐related calcium sensor protein (*Oryza sativa*)	Regulatory	1.12	**0.20**	1.28	−*Qfhs.ifa‐5A* (8 hai)	
TaAffx.616.2.S1_s_at	Hypothetical protein (Jia *et al*., 2009)	Unknown	1.49	**0.27**	1.16		
TaAffx.112045.1.S1_x_at	Glutathione‐*S*‐transferase (*Oryza sativa*)	Defence	**3.26**	1.58	1.11	+ *Qfhs.ifa‐5A* (8 hai) −*Qfhs.ifa‐5A* (72 hai)	
Ta.5004.1.S1_at	Thiol protease SEN102 precursor (*Oryza sativa*)	Metabolism	**0.29**	1.52	0.83	−*Qfhs.ifa‐5A* (8 hai)	
Ta.5004.3.S1_a_at	Thiol protease SEN102 precursor (*Oryza sativa*)	Metabolism	**0.41**	1.24	0.79	−*Qfhs.ifa‐5A* (8 hai)	
TaAffx.106424.1.S1_at	Acid phosphatase‐like (Jia *et al*., 2009)	Metabolism	0.94	1.13	**0.49**	−*Qfhs.ifa‐5A* (8 hai)	
Ta.8866.1.S1_at	Protease inhibitor‐like protein (Jia *et al*., 2009)	Defence	1.03	0.95	**0.44**	−*Qfhs.ifa‐5A* (8 hai)	
Ta.25754.1.A1_at	Glutathione‐*S*‐transferase (*Oryza sativa*)	Defence	0.99	0.96	**0.39**	−*Qfhs.ifa‐5A* (8 hai)	
Ta.1811.1.S1_at	*cis*‐Zeatin O‐glucosyltransferase (*Oryza sativa*)	Putative trichothecene detoxification and transport	0.94	0.89	**0.39**	−*Qfhs.ifa‐5A* (8 hai)	
Ta.10583.1.A1_at	tRNA synthetases class II domain‐containing protein (*Oryza sativa*)	Metabolism	0.90	1.02	**0.38**	−*Qfhs.ifa‐5A* (8 hai)	1AS, 1BS, 1 DS
TaAffx.113333.2.S1_at	Putative uncharacterized protein Sb04g011090 (*Sorghum bicolor*) (UniRef90_C5Y052)	Metabolism	0.93	1.15	**0.36**	−*Qfhs.ifa‐5A* (8 hai)	1AL, 1BL, 1DL, 5DL
Ta.30866.1.S1_at	Expressed protein‐RZ53 family‐2 (*Oryza brachyantha*) (UniRef90_B9V0I1)	Unknown	0.94	1.04	**0.36**	−*Qfhs.ifa‐5A* (8 hai)	
Ta.3545.1.S1_at		Unknown	0.98	1.04	**0.30**	−*Qfhs.ifa‐5A* (72 hai)	3AL, 3BL, 3DL
Ta.24732.1.S1_at		Unknown	1.20	1.21	**0.16**	−*Qfhs.ifa‐5A* (8 hai)	1BL, 3BL, 6DL, 7BL
Ta.23324.1.S1_at		Unknown	0.94	1.13	**0.02**		

a The expression ratio was calculated from the *F. graminearum*/mock response observed in NIL2 (resistant *Fhb1* allele) and the response provided by NIL4 (susceptible alleles at *Fhb1*).

b Annotations were assigned if *E* from the BlastX results was ≤10^−10^; otherwise, the transcripts were denominated as ‘unknown’.

c Differences in response are the means of three biological replicates. Statistically significant values are given in bold type.

d ‐/+ indicates significant positive/negative differences in response for the indicated contrast.

e Map locations were identified by searching the mapped expressed sequence tag (EST) database of Graingenes for the probe set sequences using BlastN and *E* ≤ 10^−10^.

Fifty‐four transcripts exhibited constitutive *Fhb1*‐dependent differences in abundance, when comparing mock‐inoculated samples of NIL2 and NIL4. Most transcripts showed a consistent expression pattern throughout all time points (Table S5, see Supporting Information). Of these 54 transcripts, 17 were constitutively more abundant in the presence of the resistant *Fhb1* allele (Table 2). These transcripts encode, for instance, an extensin‐like cell wall protein, a wax synthase, a calmodulin‐related calcium sensor protein, a subtilisin–chymotrypsin inhibitor (only detected for 24 hai), two seed maturation proteins and 11 proteins of unknown function.

**Figure 2:**
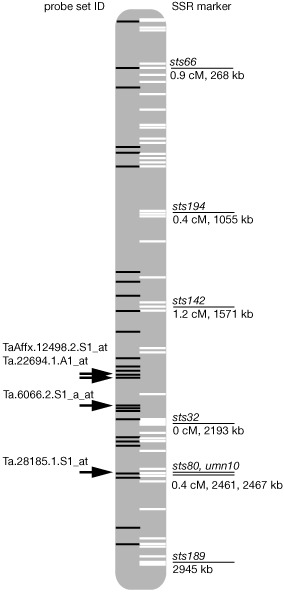
Comparison of transcripts with the physical map of the *Fhb1* locus. Simple sequence repeat (SSR) markers mapped to the sequence of the Chinese Spring BAC library contig ctg0954b of chromosome 3BS are given with genetic distances to each other and their physical position. White bars represent positions of annotated genes. Black bars represent mapped expressed sequence tag (EST) sequences corresponding to the Affymetrix wheat GeneChip. Probe set IDs of transcripts differentially changed for *Fhb1* are indicated.

To identify Affymetrix probe sets mapping in close vicinity to the physical positions of markers linked to *Fhb1*, we employed the available partial genomic sequence of chromosome 3B of cv. Chinese Spring (Choulet *et al*., 2010), which included the region harbouring *Fhb1*. We located markers *sts32*, *sts66*, *sts194*, *sts142*, *sts80*, *sts189* and *umn10* (Liu *et al*., 2006, 2008) on ctg0954b of 3BS (GenBank accession FN564434, Fig. 2). The markers span a distance of 2.94 megabases (*sts66* to *sts189*) and the region comprising the putative *Fhb1* region spans about 274 kb (*sts32* to *umn10*). From the genes represented on the 61 K wheat GeneChip, we mapped 35 with at least 98% identity to ctg0954b. Four of the 35 mapped transcripts were changed significantly for *Fhb1* in our experiments, including TaAffx.12498.2.S1_at and Ta.22694.1.A1_at (constitutively more abundant for the resistant *Fhb1* allele, both unknown) and Ta.28185.1.S1_at and Ta.6066.2.S1_a_at (constitutively more abundant for the susceptible allele, splicing factor and unknown, respectively; Fig. 2). The majority of transcripts changed for *Fhb1* could not be mapped to the interval. Only eight GeneChip probe sets correspond to the 54 annotated genes on the contig.

### Interaction effects between both QTLs identify additional *Fhb1*‐associated candidates

We also investigated whether the combined QTLs in NIL1 exhibited interaction effects between *Fhb1* and *Qfhs.ifa‐5A* when compared with the transcript accumulation observed for single QTL contrasts. These were calculated by subtracting the *F. graminearum*/mock contrasts observed in NIL2 (*Fhb1*) and NIL3 (*Qfhs.ifa‐5A*) from the contrast observed in NIL1 (both resistant alleles; interactions = NIL1 – NIL2 – NIL3 + NIL4; adjusted *P* ≤ 0.05). The pathogen‐responsive and constitutively changed transcripts are given in Tables S6 and S7, respectively (see Supporting Information).

Twenty‐three of the *F. graminearum*‐responsive transcripts that showed interaction effects at 72 hai were similarly abundant at high levels in both lines lacking the 5A resistance (NIL2 and NIL4). Hence, the interaction contrast is generated by the difference between NIL1 and NIL3, indicating potential *Fhb1* dependence (for instance, Ta.12887.1.S1_at; Fig. 1C). We tested whether these genes were also changed significantly for *Fhb1* in the genetic background with the resistant *Qfhs.ifa‐5A* allele by calculating the difference in response to *F. graminearum* between NIL1 and NIL3 (which also includes potential interaction effects; Table S8, see Supporting Information). This contrast identified the same three UGTs, two GSTs, two P450 monooxygenases and a laccase protein, as well as three metabolism‐related proteins and four unknown. The annotated gene with the highest difference in response was TaAffx.111585.1.S1. A similar contrast was also generated for *Qfhs.ifa‐5A* in the background of *Fhb1* (NIL1 – NIL2; Table S9, see Supporting Information), which, however, did not yield additional candidates.

### The *Fhb1*‐associated TaUGT12887 confers weak resistance to DON

BlastN analysis identified TaAffx.111585.1.S1_at as well as Ta.12887.1.S1_at as close homologues to *HvUGT13248*, a barley gene encoding a UGT with a proven ability to inactivate DON (Schweiger *et al*., 2010). As resistance conferred by *Fhb1* has been shown previously to be correlated with greater ability to glucosylate DON (Lemmens *et al*., 2005), both probe sets were attractive starting points for further investigations. Reconstruction of the gene models using the publically available genomic 454 sequences (http://www.cerealsdb.uk.net) of cv. Chinese Spring showed that the ESTs corresponding to both probe sets originated from the same gene. The assembled gene model contained two introns, which separated a large first exon (705 bp) and two smaller exons of 385 and 338 bp in length. The encoded open reading frame (ORF) (1428 bp) was designated *TaUGT12887* (GenBank accession JX624788). We used a collection of nulli‐tetra and ditelosomic lines to map *TaUGT12887* to chromosome 5BL. Resequencing of PCR products from both parental genotypes of the mapping population showed that the coding sequence was identical in Chinese Spring and the two parental lines used in this study, CM‐82036 and Remus.

**Figure 3:**
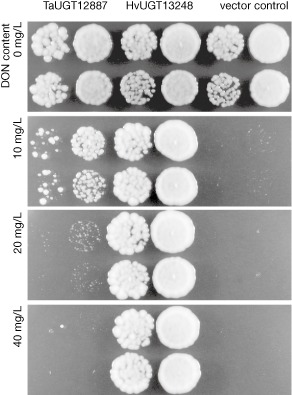
Functional evaluation of the *Fhb1*‐responsive uridine diphosphate‐glycosyltransferase (UGT) TaUGT12887. Spottings of toxin‐sensitive yeast strains expressing *TaUGT12887*, *HvUGT13248* as a positive control or the empty plasmid as negative control. Two individual transformants of each strain were spotted in different dilutions on medium containing increasing amounts of toxin. DON, deoxynivalenol.

To test whether *TaUGT12887* encodes a protein with the ability to inactivate DON, we recoded the sequence for optimal expression in yeast. The toxin‐sensitive *Saccharomyces cerevisiae* strain YZGA515 was transformed with a plasmid containing the custom synthesized ORF under the control of the strong constitutive *ADH1* promoter. Spotting assays showed that TaUGT12887 conferred only weak resistance against DON compared with the barley DON‐inactivating HvUGT13248 (Fig. 3), although the proteins were highly expressed in cell extracts, as confirmed by immunodetection of the N‐terminal c‐Myc tag (Fig. S3, see Supporting Information).

## Discussion

We investigated the effect of the *Qfhs.ifa‐5A* QTL on gene expression and reinvestigated the host response conferred by *Fhb1* using NILs harbouring either, both or none of the resistance alleles of *Fhb1* and *Qfhs.ifa‐5A*. The NILs have the European spring wheat cultivar Remus as the recurrent parent. Previous reports have shown that the impact of both QTLs is highly dependent on the genetic background of the recurrent parent (von der Ohe *et al*., 2010; Salameh *et al*., 2011). In the present study, we observed that *Qfhs.ifa‐5A* (type I resistance) reduces disease severity at a similar level to lines with *Fhb1* (type II resistance) after point inoculation—a method more sensitive to the assessment of type II resistance. The high level of resistance conferred by *Qfhs.ifa‐5A* may have been more evident with the cautious single floret inoculation method used. Most of the *F. graminearum*‐responsive genes found represent a general response to the pathogen, regardless of resistance level and QTL.

Previous transcriptomic studies on the *F. graminearum*–wheat interaction have led to the characterization of multiple defence‐associated gene classes that describe a general or genotype‐specific response of wheat to *F. graminearum* or DON (Ansari *et al*., 2007; Bernardo *et al*., 2007; Diethelm *et al*., 2012; Ding *et al*., 2011; Foroud *et al*., 2011; Golkari *et al*., 2009; Gottwald *et al*., 2012; Steiner *et al*., 2009; Walter *et al*., 2008). Walter *et al*. (2008) examined the gene expression of DON‐treated doubled haploid (DH) lines segregating for *Fhb1*, and reported the *Fhb1*‐dependent upregulation of several genes. Jia *et al*. (2009) investigated a NIL pair differential for the resistant/susceptible allele of *Fhb1*. They found 14 transcripts exhibiting a differential expression after comparing *F. graminearum*‐inoculated lines directly. Of these 14 transcripts, we identified five in our study: Ta.23324.1.S1_at (unknown), Ta.22694.1.A1_at (unknown), Ta.28185.1.S1_at (splicing factor), Ta.438.3.S1_at (nuclease) and Ta.9489.1.S1_at (regulatory gene). In addition, only a few genes identified here as QTL specific were identified in other comparable studies also using the Affymetrix GeneChip. The *F. graminearum*‐responsive Ta.24715.1.S1_at, encoding a peroxidase, was more abundant for *Fhb1* at the earliest observed time point of 8 hai and was also found to be upregulated in response to DON, but only for the susceptible genotype Superb (24 hai; Foroud *et al*., 2011). This peroxidase might play a role in the generation of reactive oxygen species (ROS). Ding *et al*. (2011) also observed earlier abundance of peroxidases in the resistant Wangshuibai compared with a susceptible mutant, and speculated that the production of ROS induces JA and ET biosynthesis (which was not observed for *Fhb1* in this work). In addition, Xiao *et al*. (2013) reported JA signalling to be upregulated for the QTL.

The complex data resulting from transcriptomic studies may become clearer if potential biochemical mechanisms are also considered. A reasonable basis for resistance to initial infection could be a result of a preformed barrier or antifungal protein or plant metabolite. From the candidates listed in Table 2, the 50–70‐fold more highly expressed LTP is the obvious candidate gene. With regard to *Fhb1*, a strong case has been made that the spread of resistance is caused by resistance to the toxin DON, which is necessary for the spread of *Fusarium*.

### Potential links to *Fhb1*‐regulated DON detoxification

Although, in the highly resistant CM‐82036, the spread of the fungus is impeded in the initially inoculated spikelet, in the NILs, the fungus manages to overwhelm resistance responses and infect the rachis, where cell wall appositions create a barrier against spread to the next spikelet. The synthesis of phenylpropanoids seems to play an important role (Gunnaiah *et al*., 2012), but the fungus seems able to inhibit its formation by the production of the protein biosynthesis inhibitor DON (Jansen *et al*., 2005). DON detoxification is not necessarily constitutive, as multiple UGTs have been shown to be inducible by DON (Gardiner *et al*., 2010; Poppenberger *et al*., 2003; Schweiger *et al*., 2013). Thus, rapid and high inducibility of such UGTs, before the toxin can block protein synthesis, may be required for resistance. Even NIL1 had, on average, four infected spikelets, indicating that the fungus can overwhelm resistance mechanisms in the inoculated spikelet. Presumably, in the more susceptible lines NIL2 and NIL4, the resistance mechanisms in the sampled inoculated spikelets have already been overwhelmed and QTL‐specific differences are no longer evident. We found one candidate UGT, designated TaUGT12887, in the *Fhb1* background, but only if basal resistance, caused by the presence of *Qfhs.ifa‐5A*, is already high (Fig. 1C). Potentially, interaction effects between both QTLs could also play a role in the greater abundance of this gene. Yet, when expressed in toxin‐sensitive yeast, TaUGT12887 only exhibits weak activity against DON compared with HvUGT13248 from barley (Schweiger *et al*., 2010).

In *Brachypodium distachyon*, all members of the orthologous gene cluster to *TaUGT12887* were found to be induced by DON, but only two of six of these genes coded for the ability to inactivate the toxin (Schweiger *et al*., 2013). A similar situation can be expected for the orthologous gene families on the three genomes of wheat, wherein *TaUGT12887* might be a DON‐responsive, but weakly active UGT, whereas yet uncharacterized paralogues, potentially not represented on the GeneChip, perform the primary detoxification function.

The formation of glutathione adducts may also contribute to DON detoxification. One GST in our data exhibited a positive difference in response to *F. graminearum* inoculation for *Fhb1* (8 hai, TaAffx.112045.1.S1_x_at): This probe set corresponds to a putative tau‐class GST, which was also identified by other contrasts. Two further GSTs (TaAffx.128514.1.S1_at and Ta.886.2.S1_at, 72 hai) were identified in the additional *Fhb1* contrast, in the background of *Qfhs.ifa‐5A*. Transcriptomic studies in DON‐infected barley indicate that the *F. graminearum* virulence factor DON most probably depletes the glutathione pool. The involvement of GSTs in the detoxification of DON is still unproven, but the nonenzymatic formation of DON–glutathione adducts has been shown (Gardiner *et al*., 2010). Recently, evidence for the occurrence *in planta* of a DON–glutathione conjugate and derived processing products in wheat has been demonstrated (Kluger *et al*., 2012).

Lemmens *et al*. (2005) have proposed that the *Fhb1* locus either encodes a UGT or a regulatory gene affecting detoxification capability, but neither the UGT nor the GST found to be differentially expressed in this study is located in the *Fhb1* interval. With regard to a potential regulator, a gene of unknown function, Ta.22694.1.A1_at, is interesting. It is constitutively more abundant (about 30‐fold) at all observed time points in the line harbouring the resistant *Fhb1* allele. This gene is located close to the predicted location of *Fhb1* between *sts142* and *sts32* (see Fig. 2). No homologous DNA is present in *Sorghum bicolor*, rice or *B. distachyon*. Only three ESTs corresponding to this sequence, two sense transcripts (cv. Chinese Spring and cv. Stephens)—for which the probe sets have been designed—and one antisense transcript (cv. Chinese Spring), are present in the database. It is unclear whether there is a meaningful (short) ORF or whether the transcript has a regulatory role.

### A constitutively more highly expressed LTP associated with *Qfhs.ifa‐5A*

We identified 338 genes exhibiting a negative difference in response for *F. graminearum* at 72 hai for *Qfhs.ifa‐5A*, indicating higher accumulation in the presence of the susceptible *Qfhs.ifa‐5A* allele. One hypothesis that explains the first counterintuitive observation of a higher upregulation of multiple defence‐related genes in the susceptible cultivar is that the fungal biomass is higher in the lines lacking the *Qfhs.ifa‐5A* resistance allele. Consequently, more plant tissue is undergoing a defence response at a given time.

Of the few candidate genes for *Qfhs.ifa‐5A* proposed in the literature, none has been identified in this study. Steiner *et al*. (2009), using a cDNA‐amplified fragment length polymorphism (AFLP) approach, investigated the QTLs in lines harbouring both *Fhb1* and *Qfhs.ifa‐5A*, and identified transcripts encoding a UGT and a PAL upregulated at 6 hai. Neither of these genes has been identified as specific for either QTL in our study.

A promising *Qfhs.ifa‐5A* candidate gene is Ta.1282.4.S1_at, a type 1 nonspecific LTP. This gene is constitutively more abundant in lines with the *Qfhs.ifa‐5A* resistant allele, exhibiting one of the largest differences in abundance in the microarray data. The position of Ta.1282.4.S1_at at 5AL, in close proximity to the centromere, is in the current confidence interval for *Qfhs.ifa‐5A* (Buerstmayr *et al*., 2003). Foroud *et al*. (2011) also reported Ta.1282.4.S1_at to be constitutively more abundant in a DH line with CYMMIT11, a FHB resistance donor with an unknown resistance QTL in its pedigree. However, screening for *Qfhs.ifa‐5A* using the flanking markers *Gwm293* and *Gwm156* did not identify *Qfhs.ifa‐5A* in their studied genotypes. The same LTP gene identified in this study was found to be associated with *Qfhs.ifa‐5A* in a microarray study comparing lines from a cross of Wuhan1 and NuyBay (T. Ouellet, Agriculture and Agri‐Food Canada, Ottawa, personal communication).

Multiple roles in defence against pathogens are ascribed to LTPs (Yeats and Rose, 2008). Sun *et al*. (2008) demonstrated that a different recombinant LTP protein from wheat can inhibit the *in vitro* growth of multiple fungal wheat pathogens, including *F. graminearum*. The *Arabidopsis thaliana DIR1* gene encodes an LTP that binds to lipids and elicits long‐distance signalling, and thus contributes to systemic acquired resistance (Maldonado *et al*., 2002). Another member of the large wheat LTP family has also been shown to compete for plasma membrane receptors with elicitins, proteins secreted by fungal pathogens from the *Phytophthora* or *Pythium* genera that elicit hypersensitive responses (Buhot *et al*., 2001). The early interaction of *F. graminearum* and the host is characterized by the release of lytic enzymes which penetrate directly the outer epidermal layers (Kang and Buchenauer, 2002). The constitutively higher abundance of LTP, also reported to act in cutin deposition (Yeats *et al*., 2010), might coincide with the type I resistance against initial fungal penetration attributed to *Qfhs.ifa‐5A*. LTPs might also contribute to toxin resistance, as the overexpression of an *A. thaliana* LTP leads to higher resistance to the trichothecene toxin trichothecin (McLaughlin *et al*., 2011). Further work to functionally characterize this LTP by stable overexpression in wheat is planned. If the role of the LTP as an *F. graminearum* resistance determinant can indeed be confirmed, new questions related to food safety must be addressed. Many PR‐14 class LTPs do not only have a role in plant defence, but are also relevant food allergens (Palacin *et al*., 2007).

Taken together, this study presents candidate genes associated with the *F. graminearum* resistance QTLs *Fhb1* and *Qfhs.ifa‐5A* and functionally validates an *Fhb1*‐related UGT, which improves resistance to DON in toxin‐sensitive yeast to a limited extent. With the release of the wheat genomic sequence in the foreseeable future, these candidates and available transcriptional data can be mapped and compared with the draft sequence of the (susceptible) cultivar Chinese Spring. Positional cloning efforts in resistant germplasm, combined with functional validation of candidates in wheat using TILLING (targeting induced local lesions in genomes) populations or virus‐induced gene silencing, will provide further insights into the resistance mediated by both QTLs. For *Qfhs.ifa‐5A*, additional efforts are required to resolve the locus in the centromeric region of chromosome 5A.

## Experimental Procedures

### Development of NILs

NILs were generated via five backcrosses to cv. Remus (FHB susceptible, Sappo/Mex//Famos) of a DH line identified from a CM‐82036 (FHB resistant, Sumai3/Thornbird) by Remus population. Selfed F_2_ progeny of BC_5_ plants were screened for the presence of the homozygous resistance QTL. NIL1 possessing the resistance alleles of *Fhb1* and *Qfhs.ifa‐5A*, NIL2 possessing *Fhb1*, NIL3 carrying the *Qfhs.ifa‐5A* resistance allele and NIL4 carrying the susceptible alleles at *Fhb1* and *Qfhs.ifa‐5A* were selected (Fig. 1A). The presence of the resistance QTL allele on 3BS was verified by the diagnostic co‐segregating marker *umn10*. The genotype of *Qfhs.ifa‐5A* was verified with flanking simple sequence repeat (SSR) markers *barc186*, *gwm1057*, *barc56*, *gwm293*, *barc117* and *gwm129* on the short arm of chromosome 5A, and *barc1*, *barc180*, *barc40* and *gmw156* on the long arm of 5A. The four NILs, CM‐82036 and Remus were also genotyped with DArT markers by Triticarte Pty. Ltd. (Canberra, Australia; Akbari *et al*., 2006; Wenzl *et al*., 2004).

### Glasshouse experiments and tissue sampling

The glasshouse experiment and inoculum production were conducted as described in Steiner *et al*. (2009) with modifications. The experiment was performed in three replications and the lines were challenged by inoculation with 10 μL of a 20 000 spores/mL *F. graminearum* suspension or water (about 200 conidia). Five heads per genotype, treatment and replication were harvested at 8, 24 and 72 hai. Treated spikelets were separated into lemma, palea and the subtending section of the rachis and the reproductive tissues, immediately frozen in liquid nitrogen and stored at −80 °C. For phenotype scoring, one central spikelet (two florets) of 50 wheat heads per genotype was inoculated on the field with 100 conidia per floret, and diseased spikelets were counted after 12, 18 and 24 days. The inoculum concentration used for phenotyping allowed better differentiation of phenotypes between NILs. Statistically significant differences between all lines were calculated using pairwise *t*‐tests (adjusted *P* ≤ 0.05). The experiment was repeated twice.

### RNA preparation and microarray hybridization

Sampled lemma, palea and rachis from five heads per experimental condition were pooled and ground to a fine powder using a mixer mill (type MM 301, Retsch, Düsseldorf, Germany). RNA was extracted according to Steiner *et al*. (2009). We employed the Affymetrix wheat Genome Array (Affymetrix, Santa Clara, CA, USA) for the identification of differential transcript abundances in our samples. The generation of labelled cRNA, hybridization and data acquisition were performed at the Microarray Facility of the University of Minnesota following the standard protocol described by Boddu *et al*. (2006).

### Array and SFP analysis

Statistical analysis was performed using the R statistical analysis environment (http://www.r‐project.org) and packages of the Bioconductor suite (http://www.bioconductor.org). We used the ‘gcrma’ package (Wu *et al*., 2004), ‘vsn’ (Huber *et al*., 2002), the fitPLM function (‘affyPLM’; Bolstad, 2004) and the lmFIT function of ‘limma’ (Smyth, 2004). To identify significant differentially expressed probe sets, we calculated *P* values after correction for multiple testing, controlling the false discovery rate (Holm, 1979). Differentially expressed transcripts were detected on the basis of an empirical Bayes moderated *t*‐statistic. For the tables shown, values have arbitrarily been limited at a nominal two‐fold difference in order to present the strongest effects only. The Affymetrix CEL, DAT and EXP files have been deposited in the Plant Expression database (PLEXdb, accession TA41). SFPs were detected between the lines by applying an algorithm described by Xu *et al*. (2009).

QTL‐specific, constitutive and *F. graminearum*‐responsive transcripts (adjusted *P* ≤ 0.05) at 8, 24 and 72 hai were calculated by subtracting the genetic background provided by NIL4 (susceptible alleles at *Fhb1* and *Qfhs.ifa‐5A*) from NIL2 (*Fhb1*) and NIL3 (*Qfhs‐ifa.5A*). In addition, we generated contrasts from NIL1 and NIL2/NIL3 to yield QTL‐specific contrasts in the background of the respective other QTL resistance allele. For the identification of transcripts exhibiting constitutive expression changes, mock‐inoculated samples were compared, whereas, for the *F. graminearum* response, transcript abundances of mock samples were subtracted from those of *F. graminearum*‐inoculated samples.

Interactions of the two QTLs were assessed by subtracting abundance differences of *F. graminearum*‐responsive transcripts identified in the single QTL contrasts (*Fhb1*, NIL2 – NIL4; *Qfhs.ifa‐5A*, NIL3 – NIL4) from the abundance differences observed in the contrast between the double QTL line NIL1 relative to NIL4. Similarly, we calculated the interaction effects comparing mock‐inoculated samples. The average *F. graminearum* response was calculated as the mean *F. graminearum* response of all four investigated genotypes.

### Annotations, mapping and resequencing of Ta.1282.4.S1_at and *TaUGT12887*

Annotations were obtained from the wheat HarvEST‐database (http://harvest.ucr.edu, Version 1.57). Ontologies, UniProt annotations and EC numbers were acquired with the Blast2GO web tool (http://www.blast2go.org). Genes were considered to be ‘unknown’ if none of the search results exhibited *E* ≤ 10^−10^. Mapping positions to the identified gene transcripts were assigned on the basis of BlastN search results against the mapped EST database hosted on the Graingenes website (http://wheat.pw.usda.gov/GG2). Moreover, we mapped transcripts (>98% identity) to the wheat contig ctg0954b, which harbours *Fhb1* (FN564434.1; Choulet *et al*., 2010) using GMAP (http://research‐pub.gene.com/gmap; Wu and Watanabe, 2005).

Selected candidates were mapped using a collection of Chinese Spring nulli‐tetra and ditelosomic lines and deletion lines of chromosome 5A (Wheat Genetics Resource Center, Kansas State University, Manhattan, KS, USA). Ta.1282.4.S1_at and *TaUGT12887* were sequenced from cDNA samples, and genomic DNA from parental cultivars CM‐82036 and Remus. All primers are given in Table S10 (see Supporting Information).

### Heterologous expression and functional testing of TaUGT12887 in *S. cerevisiae*

The coding sequence of *TaUGT12887* was optimized for expression in *S. cerevisiae* by reverse translating the amino acid sequence (according to *S. cerevisiae* codon tables provided by the Kazusa DNA Research Institute, Chiba, Japan). The gene was custom synthesized (Eurofins MWG Operon, Ebersberg, Germany) including flanking 5′ *Hind*III and 3′ *Not*I restriction sites, which were used to clone the ORF into the Hind*III*/Not*I* cloning sites of yeast expression vector pYAK7 in frame behind the N‐terminal c‐Myc epitope tag (2μ‐*P_ADH1_‐c‐Myc‐MCS‐T_ADH1_*, *LEU2*; Poppenberger *et al*., 2003). Yeast strain YZGA515 (MATa leu2‐Δ*1 trp1*‐Δ*63 ura3‐52 his3*‐Δ*200 lys2‐801 ade2‐101 pdr5::TRP1 pdr10::hisG pdr15:loxP‐KANR‐loxP ayt1::URA*) was transformed with the resulting construct, pWS60, and also with pWS1921, harbouring the previously reported DON‐UGT *HvUGT13248* (Schweiger *et al*., 2010) and the empty vector (pBP910, Poppenberger *et al*., 2003), as positive and negative controls, respectively.

Crystallized DON was dissolved in water to a stock concentration of 10 g/L. Yeast transformants were selected on synthetic complete medium lacking leucine, and grown in liquid selective medium to the exponential growth phase [optical density at 600 nm (OD_600_) = 0.7]. Three microlitres of liquid culture dilutions (OD_600_ = 0.05 and 0.005) were spotted on plates with yeast extract–peptone–dextrose (YPD) medium containing increasing amounts of DON, and incubated for 3 days at 30 °C. Immunodetection was performed with a primary mouse anti‐c‐Myc antibody (1:5000, clone 9E10; Invitrogen, Carlsbad, CA, USA) and a horseradish peroxidase (HRP)‐conjugated secondary anti‐mouse antibody (Jackson, West Grove, PA, USA). For signal detection, we used the SuperSignal West Pico chemiluminescent substrate (Pierce, Rockford, IL, USA).

### RT‐qPCR validation

A compiled list of all primers and corresponding transcripts is given in Table S11 (see Supporting Information). One microgram of total RNA was subjected to reverse transcription (QuantiTect Reverse Transcription Kit; Qiagen, Hilden, Germany). RT‐qPCRs were performed for 45 cycles in triplicate using 1 μL of cDNA and primers (300 nm in assay) in combination with the MESA GREEN qPCR Mastermix Plus (Eurogentec, Seraing, Belgium). The reference genes *glyceraldehyde‐3‐phosphate dehydrogenase* (*GAPDH*), *transcription enhancer factor‐1* (*TEF1*) and *ubiquitin* were used for normalization. Calculations of the relative fold changes were conducted as described in Pfaffl (2001). Transcripts with Ct values >40 cycles were considered as not present; the corresponding expression ratios were displayed as >100‐fold expressed.

### Metabolite extraction and high‐performance liquid chromatography‐tandem mass spectrometry (HPLC‐MS/MS) analysis

Five‐hundred milligrams of ground plant material were extracted by the addition of 4 mL of solvent consisting of 79% acetonitrile, 1% acetic acid and 20% water. Samples were vigorously shaken at room temperature for 2 h, spun down and the clear supernatant was subjected to HPLC‐MS/MS analysis. *Fusarium graminearum*‐treated samples of NILs at 72 hai were analysed for DON and D3G (Berthiller *et al*., 2005).

## Supporting information


**Fig. S1** Reverse transcription‐quantitative polymerase chain reaction (RT‐qPCR) confirmation of selected transcripts.

**Fig. S2** Multiple alignment of the resequenced wheat lipid transfer proteins (LTPs) corresponding to Ta.1282.4.S1_at in cv. CM‐82036 and Remus.

**Fig. S3** Immunoblotting of the N‐terminal c‐Myc antibody used to detect uridine diphosphate‐glycosyltransferase (UGT) expression levels (A) and the corresponding Coomassie‐stained acryl amide gel as loading control (B).

**Table S1** Number of transcripts exhibiting a significant difference in response to *Fusarium graminearum* for either *Fhb1* or *Qfhs.ifa‐5A* at the given time point.

**Table S2** Functional classification of *Fusarium graminearum*‐responsive transcripts and transcripts showing constitutive quantitative trait locus (QTL)‐specific differences in transcript abundance (adjusted *P* ≤ 0.05, ≥2 fold change).

**Table S3** Transcripts exhibiting a negative difference in response for the resistant allele of *Qfhs.ifa‐5A* (adjusted *P* ≤ 0.05, ≥2 fold change, FC) after inoculation with *Fusarium graminearum* spores at 72 h after inoculation.

**Table S4** Transcripts exhibiting constitutive differences in transcript abundance for *Qfhs.ifa‐5A* (adjusted *P* ≤ 0.05, ≥2 fold change, FC).

**Table S5** Transcripts exhibiting constitutive differences in transcript abundance for *Fhb1* (adjusted *P* ≤ 0.05, ≥2 fold change, FC).

**Table S6** *Fusarium graminearum*‐responsive transcripts exhibiting nonadditive effects between *Fhb1* and *Qfhs.ifa‐5A* (adjusted *P* ≤ 0.05, ≥2 fold change, FC).

**Table S7** Transcripts exhibiting nonadditive effects between *Fhb1* and *Qfhs.ifa‐5A* (adjusted *P* ≤ 0.05, ≥2 fold change, FC) in mock‐inoculated samples.

**Table S8** Transcripts exhibiting a significant difference in response between NIL1 and NIL3 yielding *Fhb1*‐related transcripts in the presence of the *Qfhs.ifa‐5A* resistance allele (adjusted *P* ≤ 0.05, ≥2 fold change, FC) after inoculation with *Fusarium graminearum* spores.

**Table S9** Transcripts exhibiting a significant difference in response between NIL1 and NIL2 yielding *Qfhs.ifa‐5A*‐related transcripts in the presence of the *Fhb1* resistance allele (adjusted *P* ≤ 0.05, ≥2 fold change, FC) after inoculation with *Fusarium graminearum* spores.

**Table S10** Sequences of primers used for sequencing and mapping of Ta.1282.4.S1_at and *TaUGT12887*.

**Table S11** Sequences of primers used for the validation of transcript abundances by reverse transcription‐quantitative polymerase chain reaction (RT‐qPCR).

## References

[mpp12048-bib-0001] Akbari, M. , Wenzl, P. , Caig, V. , Carling, J. , Xia, L. , Yang, S. , Uszynski, G. , Mohler, V. , Lehmensiek, A. , Kuchel, H. , Hayden, M.J. , Howes, N. , Sharp, P. , Vaughan, P. , Rathmell, B. , Huttner, E. and Kilian, A. (2006) Diversity arrays technology (DArT) for high‐throughput profiling of the hexaploid wheat genome. Theor. Appl. Genet. 113, 1409–1420. 17033786 10.1007/s00122-006-0365-4

[mpp12048-bib-0002] Anderson, J. , Stack, R. , Liu, S. and Waldron, B. (2001) DNA markers for Fusarium head blight resistance QTLs in two wheat populations. Theor. Appl. Genet. 102, 1164–1168.

[mpp12048-bib-0003] Ansari, K.I. , Walter, S. , Brennan, J.M. , Lemmens, M. , Kessans, S. , McGahern, A. , Egan, D. and Doohan, F.M. (2007) Retrotransposon and gene activation in wheat in response to mycotoxigenic and non‐mycotoxigenic‐associated Fusarium stress. Theor. Appl. Genet. 114, 927–937. 17256175 10.1007/s00122-006-0490-0

[mpp12048-bib-0004] Bernardo, A. , Bai, G. , Guo, P. , Xiao, K. , Guenzi, A.C. and Ayoubi, P. (2007) *Fusarium graminearum*‐induced changes in gene expression between Fusarium head blight‐resistant and susceptible wheat cultivars. Funct. Integr. Genomics, 7, 69–77. 16636822 10.1007/s10142-006-0028-1

[mpp12048-bib-0005] Berthiller, F. , Dall'Asta, C. , Schuhmacher, R. , Lemmens, M. , Adam, G. and Krska, R. (2005) Masked mycotoxins: determination of a deoxynivalenol glucoside in artificially and naturally contaminated wheat by liquid chromatography‐tandem mass spectrometry. J. Agric. Food Chem. 53, 3421–3425. 15853382 10.1021/jf047798g

[mpp12048-bib-0006] Boddu, J. , Cho, S. , Kruger, W.M. and Muehlbauer, G.J. (2006) Transcriptome analysis of the barley–*Fusarium graminearum* interaction. Mol. Plant–Microbe Interact. 19, 407–417. 16610744 10.1094/MPMI-19-0407

[mpp12048-bib-0007] Bolstad, B.M. (2004) Low‐level analysis of high‐density oligonucleotide array data: background, normalization and summarization. PhD Dissertation. Berkeley, CA: University of California.

[mpp12048-bib-0008] Boutrot, F. , Meynard, D. , Guiderdoni, E. , Joudrier, P. and Gautier, M.‐F. (2007) The *Triticum aestivum* non‐specific lipid‐transfer protein (TaLtp) gene family: comparative promoter activity of six TaLtp genes in transgenic rice. Planta, 225, 843–862. 16983534 10.1007/s00425-006-0397-7

[mpp12048-bib-0010] Buerstmayr, H. , Lemmens, M. , Hartl, L. , Doldi, L. , Steiner, B. , Stierschneider, M. and Ruckenbauer, P. (2002) Molecular mapping of QTLs for Fusarium head blight resistance in spring wheat. I. Resistance to fungal spread (type II resistance). Theor. Appl. Genet. 104, 84–91. 12579431 10.1007/s001220200009

[mpp12048-bib-0011] Buerstmayr, H. , Steiner, B. , Hartl, L. , Griesser, M. , Angerer, N. , Lengauer, D. , Miedaner, T. , Schneider, B. and Lemmens, M. (2003) Molecular mapping of QTLs for Fusarium head blight resistance in spring wheat. II. Resistance to fungal penetration and spread. Theor. Appl. Genet. 107, 503–508. 12768240 10.1007/s00122-003-1272-6

[mpp12048-bib-0009] Buerstmayr, H. , Ban, T. and Anderson, J.A. (2009) QTL mapping and marker‐assisted selection for Fusarium head blight resistance in wheat: a review. Plant Breed. 128, 1–26.

[mpp12048-bib-0012] Buhot, N. , Douliez, J.P. , Jacquemard, A. , Marion, D. , Tran, V. , Maume, B.F. , Milat, M.L. , Ponchet, M. , Mikès, V. , Kader, J.C. and Blein, J.P. (2001) A lipid‐transfer protein binds to a receptor involved in the control of plant defence responses. FEBS Lett. 509, 27–30. 11734200 10.1016/s0014-5793(01)03116-7

[mpp12048-bib-0013] Choulet, F. , Wicker, T. , Rustenholz, C. , Paux, E. , Salse, J. , Leroy, P. , Schlub, S. , Le Paslier, M.‐C. , Magdelenat, G. , Gonthier, C. , Couloux, A. , Budak, H. , Breen, J. , Pumphrey, M. , Liu, S. , Kong, X. , Jia, J. , Gut, M. , Brunel, D. , Anderson, J.A. , Gill, B.S. , Appels, R. , Keller, B. and Feuillet, C. (2010) Megabase level sequencing reveals contrasted organization and evolution patterns of the wheat gene and transposable element spaces. Plant Cell, 22, 1686–1701. 20581307 10.1105/tpc.110.074187 PMC2910976

[mpp12048-bib-0014] Desjardins, A.E. (2006) Fusarium Mycotoxins: Chemistry, Genetics, and Biology. St. Paul, MN: APS Press.

[mpp12048-bib-0015] Diethelm, M. , Rhiel, M. , Wagner, C. , Mikolajewski, S. , Groth, J. , Hartl, L. , Friedt, W. and Schweizer, G. (2012) Gene expression analysis of four WIR1‐like genes in floret tissues of European winter wheat after challenge with *G. zeae* . Euphytica, 186, 103–114.

[mpp12048-bib-0016] Ding, L. , Xu, H. , Yi, H. , Yang, L. , Kong, Z. , Zhang, L. , Xue, S. , Jia, H. and Ma, Z. (2011) Resistance to hemi‐biotrophic *F. graminearum* infection is associated with coordinated and ordered expression of diverse defense signaling pathways. PloS ONE, 6, e19008. 21533105 10.1371/journal.pone.0019008 PMC3080397

[mpp12048-bib-0018] Foroud, N.A. , Ouellet, T. , Laroche, A. , Oosterveen, B. , Jordan, M.C. , Ellis, B.E. and Eudes, F. (2011) Differential transcriptome analyses of three wheat genotypes reveal different host response pathways associated with Fusarium head blight and trichothecene resistance. Plant Pathol. 61, 296–314.

[mpp12048-bib-0019] Gardiner, S. , Boddu, J. , Berthiller, F. , Hametner, C. , Stupar, R.M. , Adam, G. and Muehlbauer, G.J. (2010) Transcriptome analysis of the barley–deoxynivalenol interaction: evidence for a role of glutathione in deoxynivalenol detoxification. Mol. Plant–Microbe Interact. 23, 962–976. 20521958 10.1094/MPMI-23-7-0962

[mpp12048-bib-0020] Golkari, S. , Gilbert, J. , Ban, T. and Procunier, J.D. (2009) QTL‐specific microarray gene expression analysis of wheat resistance to Fusarium head blight in Sumai‐3 and two susceptible NILs. Genome, 52, 409–418. 19448721 10.1139/g09-018

[mpp12048-bib-0021] Gottwald, S. , Samans, B. , Lück, S. and Friedt, W. (2012) Jasmonate and ethylene dependent defence gene expression and suppression of fungal virulence factors: two essential mechanisms of Fusarium head blight resistance in wheat? BMC Genomics, 13, 369. 22857656 10.1186/1471-2164-13-369 PMC3533685

[mpp12048-bib-0022] Gunnaiah, R. , Kushalappa, A.C. , Duggavathi, R. , Fox, S. and Somers, D.J. (2012) Integrated metabolo‐proteomic approach to decipher the mechanisms by which wheat QTL (*Fhb1*) contributes to resistance against *F. graminearum* . PLoS ONE, 7, e40695. 22866179 10.1371/journal.pone.0040695 PMC3398977

[mpp12048-bib-0023] Holm, S. (1979) A simple sequentially rejective multiple test procedure. Scand. J. Stat. 6, 65–70.

[mpp12048-bib-0024] Horevaj, P. , Brown‐Guedira, G. and Milus, E.A. (2012) Resistance in winter wheat lines to deoxynivalenol applied into florets at flowering stage and tolerance to phytotoxic effects on yield. Plant Pathol. 61, 925–933.

[mpp12048-bib-0025] Huber, W. , von Heydebreck, A. , Sültmann, H. , Poustka, A. and Vingron, M. (2002) Variance stabilization applied to microarray data calibration and to the quantification of differential expression. Bioinformatics, 18, 96–104. 10.1093/bioinformatics/18.suppl_1.s96 12169536

[mpp12048-bib-0026] Jansen, C. , von Wettstein, D. , Schäfer, W. , Kogel, K.H. , Felk, A. and Maier, F.J. (2005) Infection patterns in barley and wheat spikes inoculated with wild‐type and trichodiene synthase gene disrupted *Fusarium graminearum* . Proc. Natl. Acad. Sci. USA, 102, 16 892–16 897. 10.1073/pnas.0508467102 PMC1283850 16263921

[mpp12048-bib-0027] Jia, H. , Cho, S. and Muehlbauer, G.J. (2009) Transcriptome analysis of a wheat near‐isogenic line pair carrying Fusarium head blight‐resistant and ‐susceptible alleles. Mol. Plant–Microbe Interact. 22, 1366–1378. 19810806 10.1094/MPMI-22-11-1366

[mpp12048-bib-0028] Kang, Z. and Buchenauer, H. (2002) Immunocytochemical localization of β‐1,3‐glucanase and chitinase in *Fusarium culmorum*‐infected wheat spikes. Physiol. Mol. Plant Pathol. 60, 141–153.

[mpp12048-bib-0029] Kluger, B. , Büschl, C. , Lemmens, M. , Häubl, G. , Labuda, R. , Adam, G. , Krska, R. and Schuhmacher, R. (2012) Stable isotopic labelling‐assisted untargeted screening reveals novel conjugates of the mycotoxin deoxynivalenol in wheat. Anal. Bioanal. Chem. 405, 5031–5036. 23086087 10.1007/s00216-012-6483-8 PMC3656241

[mpp12048-bib-0030] Lemmens, M. , Scholz, U. , Berthiller, F. , Dall'Asta, C. , Koutnik, A. , Schuhmacher, R. , Adam, G. , Buerstmayr, H. , Mesterházy, A. , Krska, R. and Ruckenbauer, P. (2005) The ability to detoxify the mycotoxin deoxynivalenol colocalizes with a major quantitative trait locus for Fusarium head blight resistance in wheat. Mol. Plant–Microbe Interact. 18, 1318–1324. 16478051 10.1094/MPMI-18-1318

[mpp12048-bib-0031] Leonard, K.J. and Bushnell, W.R. (2003) Fusarium Head Blight of Wheat and Barley. St. Paul, MN: APS Press.

[mpp12048-bib-0033] Liu, S. , Zhang, X. , Pumphrey, M.O. , Stack, R.W. , Gill, B.S. and Anderson, J. (2006) Complex microcolinearity among wheat, rice, and barley revealed by fine mapping of the genomic region harboring a major QTL for resistance to Fusarium head blight in wheat. Funct. Integr. Genomics, 6, 83–89. 16270217 10.1007/s10142-005-0007-y

[mpp12048-bib-0032] Liu, S. , Pumphrey, M.O. , Gill, B.S. , Trick, H.N. , Zhang, J.X. , Dolezel, J. , Chalhoub, B. and Anderson, J. (2008) Toward positional cloning of Fhb1, a major QTL for Fusarium head blight resistance in wheat. Cereal Res. Commun. 36, 195–201.

[mpp12048-bib-0034] Maldonado, A.M. , Doerner, P. , Dixon, R.A. , Lamb, C.J. and Cameron, R.K. (2002) A putative lipid‐transfer protein involved in systemic resistance signalling in Arabidopsis. Nature, 419, 399–403. 12353036 10.1038/nature00962

[mpp12048-bib-0035] McLaughlin, J. , Bin Umer, A. , Basu, D. , McCormick, S. and Tumer, N.E. (2011) An activation tagging screen to identify novel genes for Fusarium head blight (FHB) resistance. Proceedings of the National Fusarium Head Blight Forum. St. Louis, MO, USA, p. 90 East Lansing: US Wheat and Barley Scab Initiative.

[mpp12048-bib-0036] von der Ohe, C. , Ebmeyer, E. , Korzun, V. and Miedaner, T. (2010) Agronomic and quality performance of winter wheat backcross populations carrying non‐adapted Fusarium head blight resistance QTL. Crop Sci. 50, 2283–2290.

[mpp12048-bib-0037] Palacin, A. , Quirce, S. , Armentia, A. , Fernández‐Nieto, M. , Pacios, L.F. , Asensio, T. , Sastre, J. , Diaz‐Perales, A. and Salcedo, G. (2007) Wheat lipid transfer protein is a major allergen associated with baker's asthma. J. Allergy Clin. Immunol. 120, 1132–1138. 17716720 10.1016/j.jaci.2007.07.008

[mpp12048-bib-0038] Pestka, J.J. (2010) Deoxynivalenol: mechanisms of action, human exposure, and toxicological relevance. Arch. Toxicol. 84, 663–679. 20798930 10.1007/s00204-010-0579-8

[mpp12048-bib-0039] Pfaffl, M.W. (2001) A new mathematical model for relative quantification in real‐time RT‐PCR. Nucleic Acids Res. 29, e45. 11328886 10.1093/nar/29.9.e45 PMC55695

[mpp12048-bib-0040] Poppenberger, B. , Berthiller, F. , Lucyshyn, D. , Sieberer, T. , Schuhmacher, R. , Krska, R. , Kuchler, K. , Glössl, J. , Luschnig, C. and Adam, G. (2003) Detoxification of the Fusarium mycotoxin deoxynivalenol by a UDP‐glucosyltransferase from *Arabidopsis thaliana* . J. Biol. Chem. 278, 47 905–47 914. 10.1074/jbc.M307552200 12970342

[mpp12048-bib-0041] Salameh, A. , Buerstmayr, M. , Steiner, B. , Neumayer, A. , Lemmens, M. and Buerstmayr, H. (2011) Effects of introgression of two QTL for Fusarium head blight resistance from Asian spring wheat by marker‐assisted backcrossing into European winter wheat on Fusarium head blight resistance, yield and quality traits. Mol. Breed. 28, 485–494.

[mpp12048-bib-0042] Schweiger, W. , Boddu, J. , Shin, S. , Poppenberger, B. , Berthiller, F. , Lemmens, M. , Muehlbauer, G.J. and Adam, G. (2010) Validation of a candidate deoxynivalenol‐inactivating UDP‐glucosyltransferase from barley by heterologous expression in yeast. Mol. Plant–Microbe Interact. 23, 977–986. 20521959 10.1094/MPMI-23-7-0977

[mpp12048-bib-0043] Schweiger, W. , Pasquet, J.‐C. , Nussbaumer, T. , Kovalsky Paris, M.P. , Wiesenberger, G. , Macadré, C. , Ametz, C. , Berthiller, F. , Lemmens, M. , Saindrenan, P. , Mewes, H.‐W. , Mayer, K.F.X. , Dufresne, M. and Adam, G. (2013) Functional characterization of two clusters of *Brachypodium distachyon* UDP‐glycosyltransferases encoding putative deoxynivalenol detoxification genes. Mol. Plant–Microbe Interact. 26, 781–792. 23550529 10.1094/MPMI-08-12-0205-R

[mpp12048-bib-0044] Shen, X. , Francki, M.G. and Ohm, H.W. (2006) A resistance‐like gene identified by EST mapping and its association with a QTL controlling *Fusarium* head blight infection on wheat chromosome 3BS. Genome, 49, 631–635. 16936842 10.1139/g06-010

[mpp12048-bib-0045] Shin, S. , Torres‐Acosta, J.A. , Heinen, S.J. , McCormick, S. , Lemmens, M. , Paris, M.P. , Berthiller, F. , Adam, G. and Muehlbauer, G.J. (2012) Transgenic *Arabidopsis thaliana* expressing a barley UDP‐glucosyltransferase exhibit resistance to the mycotoxin deoxynivalenol. J. Exp. Bot. 63, 4731–4740. 22922639 10.1093/jxb/ers141 PMC3428005

[mpp12048-bib-0046] Smyth, G.K. (2004) Linear models and empirical Bayes methods for assessing differential expression in microarray experiments. Stat. Appl. Genet. Mol. Biol. 3, 3. 10.2202/1544-6115.1027 16646809

[mpp12048-bib-0047] Steiner, B. , Kurz, H. , Lemmens, M. and Buerstmayr, H. (2009) Differential gene expression of related wheat lines with contrasting levels of head blight resistance after *Fusarium graminearum* inoculation. Theor. Appl. Genet. 118, 753–764. 19082576 10.1007/s00122-008-0935-8 PMC3194064

[mpp12048-bib-0048] Sun, J.‐Y. , Gaudet, D. , Lu, Z.‐X. , Frick, M. , Puchalski, B. and Laroche, A. (2008) Characterization and antifungal properties of wheat nonspecific lipid‐transfer proteins. Mol. Plant–Microbe Interact. 21, 346–360. 18257684 10.1094/MPMI-21-3-0346

[mpp12048-bib-0017] The European Commission (2006) Commission recommendation (EC) no. 1881/2006 of 19 December 2006 setting maximum levels for certain contaminants in foodstuffs. OJ L, 364, 5–24.

[mpp12048-bib-0049] Volpi, C. , Janni, M. , Lionetti, V. , Bellincampi, D. , Favaron, F. and D'Ovidio, R. (2011) The ectopic expression of a pectin methyl esterase inhibitor increases pectin methyl esterification and limits fungal diseases in wheat. Mol. Plant–Microbe Interact. 24, 1012–1019. 21585271 10.1094/MPMI-01-11-0021

[mpp12048-bib-0050] Walter, S. , Brennan, J.M. , Arunachalam, C. , Ansari, K.I. , Hu, X. , Khan, M.R. , Trognitz, F. , Trognitz, B. , Leonard, G. , Egan, D. and Doohan, F.M. (2008) Components of the gene network associated with genotype‐dependent response of wheat to the Fusarium mycotoxin deoxynivalenol. Funct. Integr. Genomics, 8, 421–427. 18592282 10.1007/s10142-008-0089-4

[mpp12048-bib-0051] Walter, S. , Nicholson, P. and Doohan, F.M. (2010) Action and reaction of host and pathogen during Fusarium head blight disease. New Phytol. 185, 54–66. 19807873 10.1111/j.1469-8137.2009.03041.x

[mpp12048-bib-0052] Wenzl, P. , Carling, J. , Kudrna, D. , Jaccoud, D. , Huttner, E. , Kleinhofs, A. and Kilian, A. (2004) Diversity Arrays Technology (DArT) for whole‐genome profiling of barley. Proc. Natl. Acad. Sci. USA, 101, 9915–9920. 15192146 10.1073/pnas.0401076101 PMC470773

[mpp12048-bib-0053] Wu, T.D. and Watanabe, C.K. (2005) GMAP: a genomic mapping and alignment program for mRNA and EST sequences. Bioinformatics, 21, 1859–1875. 15728110 10.1093/bioinformatics/bti310

[mpp12048-bib-0054] Wu, Z. , Irizarry, R.A. , Gentleman, R. , Martinez‐Murillo, F. and Spencer, F. (2004) A model‐based background adjustment for oligonucleotide expression arrays. J. Am. Stat. Assoc. 99, 909–917.

[mpp12048-bib-0055] Xiao, J. , Jin, X. , Wang, H. , Cao, A. , Zhao, W. , Pei, H. , Xue, Z. , He, L. , Chen, Q. and Wang, X. (2013) Transcriptome‐based discovery of pathways and genes related to resistance against Fusarium head blight in wheat landrace Wangshuibai. BMC Genomics, 14, 197. 23514540 10.1186/1471-2164-14-197 PMC3616903

[mpp12048-bib-0056] Xu, W.W. , Cho, S. , Yang, S.S. , Bolon, Y.‐T. , Bilgic, H. , Jia, H. , Xiong, Y. and Muehlbauer, G.J. (2009) Single‐feature polymorphism discovery by computing probe affinity shape powers. BMC Genet. 10, 48. 19709416 10.1186/1471-2156-10-48 PMC2746803

[mpp12048-bib-0057] Yeats, T.H. and Rose, J.K.C. (2008) The biochemistry and biology of extracellular plant lipid‐transfer proteins (LTPs). Protein Sci. 17, 191–198. 18096636 10.1110/ps.073300108 PMC2222726

[mpp12048-bib-0058] Yeats, T.H. , Howe, K.J. , Matas, A.J. , Buda, G.J. , Thannhauser, T.W. and Rose, J.K. (2010) Mining the surface proteome of tomato (*Solanum lycopersicum*) fruit for proteins associated with cuticle biogenesis. J. Exp. Bot. 61, 3759–3771. 20571035 10.1093/jxb/erq194 PMC2921210

[mpp12048-bib-0059] Zhuang, Y. , Gala, A. and Yen, Y. (2013) Identification of functional genic components of major Fusarium head blight resistance quantitative trait loci in wheat cultivar Sumai3. Mol. Plant–Microbe Interact. 26, 442–450. 23234406 10.1094/MPMI-10-12-0235-R

